# Post-acute sequelae of SARS-CoV-2 associates with physical inactivity in a cohort of COVID-19 survivors

**DOI:** 10.1038/s41598-022-26888-3

**Published:** 2023-01-05

**Authors:** Saulo Gil, Bruno Gualano, Adriana Ladeira de Araújo, Gersiel Nascimento de Oliveira Júnior, Rodolfo Furlan Damiano, Fabio Pinna, Marta Imamura, Vanderson Rocha, Esper Kallas, Linamara Rizzo Batistella, Orestes V. Forlenza, Carlos R. R. de Carvalho, Geraldo Filho Busatto, Hamilton Roschel, HCFMUSP COVID-19 Study Group, Edivaldo Utiyama, Aluisio Segurado, Beatriz Perondi, Anna Miethke Morais, Amanda Montal, Leila Letaif, Solange Fusco, Marjorie Fregonesi Rodrigues da Silva, Marcelo Rocha, Izabel Marcilio, Izabel Cristina Rios, Fabiane Yumi Ogihara Kawano, Maria Amélia de Jesus, Ésper Georges Kallas, Carolina Carmo, Clarice Tanaka, Heraldo Possolo de Souza, Julio F. M. Marchini, Carlos Carvalho, Juliana Carvalho Ferreira, Maura Salaroli de Oliveira, Thaís Guimarães, Carolina dos Santos Lázari, Alberto José da Silva Duarte, Ester Sabino, Marcello Mihailenko Chaves Magri, Tarcisio E. P. Barros-Filho, Maria Cristina Peres Braido Francisco

**Affiliations:** 1 Applied Physiology and Nutrition Research Group, Laboratory of Assessment and Conditioning in Rheumatology, School of Physical Education and Sport, School of Medicine FMUSP, University of Sao Paulo, Av. Dr. Arnaldo, 455, Pacaembu, São Paulo, SP Brazil; 2 Rheumatology Division, Faculdade de Medicina FMUSP Hospital das Clinicas HCFMUSP, Universidade de Sao Paulo, São Paulo, SP Brazil; 3 Diretoria Executiva dos LIMs, Faculdade de Medicina da Universidade de São Paulo, Sao Paulo, Brazil; 4 Departamento e Instituto de Psiquiatria, Hospital das Clínicas da Faculdade de Medicina da Universidade de São Paulo HCFMUSP, São Paulo, SP Brazil; 5 Otorrhinolaringoly Division, Faculdade de Medicina, Hospital das Clinicas HCFMUSP, University of São Paulo, São Paulo, Brazil; 6 Instituto de Medicina Física e de Reabilitação, Hospital das Clínicas da Faculdade de Medicina da Universidade de São Paulo, São Paulo, Brazil; 7 Departamento de Clínica Médica, Hospital das Clínicas da Faculdade de Medicina da Universidade de São Paulo, São Paulo, Brazil; 8 Laboratório de Genética e Hematologia Molecular, Hospital das Clínicas da Faculdade de Medicina da Universidade de São Paulo, São Paulo, Brazil; 9 Departamento de Moléstias Infecciosas e Parasitárias, Faculdade de Medicina da Universidade de São Paulo, São Paulo, Brazil; 10 Departamento de Clínica Médica, Laboratório de Imunologia Clínica e Alergia, Hospital das Clínicas da Faculdade de Medicina da Universidade de São Paulo, São Paulo, Brazil; 11 Departamento de Cardio-Pneumologia, Faculdade de Medicina da Universidade de São Paulo, São Paulo, Brazil; 12 Faculdade de Medicina da Universidade de São Paulo, School of Medicine, University of Sao Paulo, São Paulo, Brazil

**Keywords:** Signs and symptoms, Risk factors

## Abstract

The aim of this study was to determine whether Post-acute Sequelae of SARS-CoV-2 Infection (PASC) are associated with physical inactivity in COVID-19 survivors. This is a cohort study of COVID-19 survivors discharged from a tertiary hospital in Sao Paulo, Brazil. Patients admitted as inpatients due to laboratory-confirmed COVID-19 between March and August 2020 were consecutively invited for a follow-up in-person visit 6 to 11 months after hospitalization. Ten symptoms of PASC were assessed using standardized scales. Physical activity was assessed by questionnaire and participants were classified according to WHO Guidelines. 614 patients were analyzed (age: 56 ± 13 years; 53% male). Frequency of physical inactivity in patients exhibiting none, at least 1, 1–4, and 5 or more symptoms of PASC was 51%, 62%, 58%, and 71%, respectively. Adjusted models showed that patients with one or more persistent PASC symptoms have greater odds of being physically inactive than those without any persistent symptoms (OR: 1.57 [95% CI 1.04–2.39], *P* = 0.032). Dyspnea (OR: 2.22 [1.50–3.33], *P* < 0.001), fatigue (OR: 2.01 [1.40–2.90], *P* < 0.001), insomnia (OR: 1.69 [1.16–2.49], *P* = 0.007), post-traumatic stress (OR: 1.53 [1.05–2.23], *P* = 0.028), and severe muscle/joint pain (OR: 1.53 [95% CI 1.08–2.17], *P* = 0.011) were associated with greater odds of being physically inactive. This study suggests that PASC is associated with physical inactivity, which itself may be considered as a persistent symptom among COVID-19 survivors. This may help in the early identification of patients who could benefit from additional interventions tailored to combat inactivity (even after treatment of PASC), with potential beneficial impacts on overall morbidity/mortality and health systems worldwide.

## Introduction

COVID-19 pandemic is raising a devastating impact on public health, resulting in millions of hospitalizations and deaths globally^1^. Among survivors, the high occurrence of patients reporting post-acute sequelae of SARS-CoV-2 (PASC) is a great cause of concern, as it threatens health systems worldwide. This condition, also known as “long COVID”, is defined as the illness that occurs in people who have a history of probable or confirmed SARS-CoV-2 infection, usually within 3 months from the onset of COVID-19, with symptoms and effects that last for at least 2 months^2^. Early reports revealed that around 76% of patients reported at least 1 persistent symptom 6 months following hospital discharge^3^, with fatigue, dyspnea, cough, headache, loss of taste or smell, and cognitive or mental health impairments (e.g., anxiety or depression) being the most commonly reported symptoms^4–7^.

Physical inactivity (i.e., < 150 min/week at moderate-to-vigorous physical activity) is widely recognized as an independent risk factor for impaired functional status^8^, musculoskeletal disorders^9^, anxiety and depression^10^, and all-cause mortality^11^. Only a single study showed that patients who experienced persistent symptoms 6 months after COVID-19 reported lower physical activity levels compared to the pre-infection period^12^. Considering the detrimental effects that physical inactivity may have upon overall health status and quality of life in COVID-19 survivors, it is of public health importance to determine the risk factors related to PASC that may predispose to physical inactivity and help to early identify individuals that are more likely to be physically inactive.

Therefore, we aimed to determine whether PASC are associated with physical inactivity in a cohort of 614 COVID-19 survivors who underwent in-person multidisciplinary assessments conducted 6–11 months following hospitalization in a tertiary hospital in Brazil.

## Results

A total of 749 eligible individuals attended the in-person follow-up assessment; 614 had complete data and were included in the analysis. Table 1 shows the characteristics of these patients. The sample comprised patients of both sexes (53% male) aged 56 ± 13 years. The frequency of low, middle, and high socioeconomic status was 9%, 50% and 40%, respectively. This is a similar profile to that of the city of Sao Paulo, according to the National Household Sample Survey (*Pesquisa Nacional por Amostra de Domicílio*—PNADC—2021) from the Brazilian Institute of Geography and Statistics^13^. Thirty-seven percent of the patients were smoking at baseline. Prevalence of current hypertension, type 2 diabetes, and obesity were 58%, 35%, and 17%, respectively. Fifty five percent of the patients required intensive care and 37% used invasive mechanical ventilation. Only 40% of the patients met the physical activity recommendations. Table 2 shows the prevalence of physical inactivity according to sex and age.

**Table 1:** Sociodemographic and clinical characteristics of patients.

	All patients (n = 614)
Age, median (range), years	56 (18–87)
**Sex, n (%)**	
Female	287 (46.7%)
Male	327 (53.3%)
**Race, n (%)**	
White	86 (14.0%)
Black	238 (38.7%)
Pardo^a^	283 (46.1%)
Asian	7 (1.2%)
**Socioeconomic status, n (%)**	
Low	57 (9.3%)
Middle	311 (50.6%)
High	246 (40.1%)
**Smoking status, n (%)**	
Never	386 (62.9%)
Current/others	228 (37.1%)
Hospital Length of Stay, median (range), days	12 (2–163)
**Pre-existing conditions, n (%)**	
Hypertension	360 (58%)
Type 2 Diabetes	215 (35%)
Obesity (BMI ≥ 30 kg/m^2^)	106 (17%)
ICU Admission, n (%)	338 (55%)
Use of Invasive Mechanical Ventilation, n (%)	231 (37%)

*BMI* Body mass index, *ICU* Intensive care unit.

^a^Pardo is the exact term used in Brazilian Portuguese, meaning “mixed ethnicity,” according to the Brazilian Institute of Geography and Statistics.

**Table 2:** Prevalence of physical inactivity according to sex and age.

Physical inactivity (< 150 min/week), n (%)	All patients (n = 614)
Total	369 (60%)
Female	176 (61%)
Male	193 (59%)
< 60 years old	195 (54%)
≥ 60 years old	174 (68%)

Prevalence of physical inactivity in patients exhibiting none, at least 1, 1–4, and 5 or more PASC symptoms were 51%, 62%, 58%, and 71%, respectively. The frequency of physical inactivity in patients reporting different PASC were: dyspnea (77%), fatigue (69%), severe muscle/joint pain (66%), insomnia (66%), post-traumatic stress disorder (65%), memory impairments (65%), anxiety (65%), taste (65%) and smell (63%) loss, and depression (62%). Table 3 details the prevalence of physical inactivity according to the presence of post-acute sequelae of SARS-CoV-2.

**Table 3:** Relative frequency of physically inactive and active individuals (> 150 min/week) according to the presence of post-acute sequelae of SARS-CoV-2 evaluated 6–11 months following hospitalization.

	Physically inactive/physically active (%)
No PASC	51/49
At least 1 symptom	62/38
1–4 symptoms	58/42
5 or more symptoms	71/29
Dyspnea	77/23
Fatigue	69/31
Severe muscle/joint pain	66/34
Insomnia	66/34
Post-traumatic stress disorder	65/35
Memory impairments	65/35
Anxiety	65/35
Taste loss	65/35
Smell loss	63/37
Depression	62/38

*PASC* Post-acute sequelae of SARS-CoV-2 infection.

**Figure 1:**
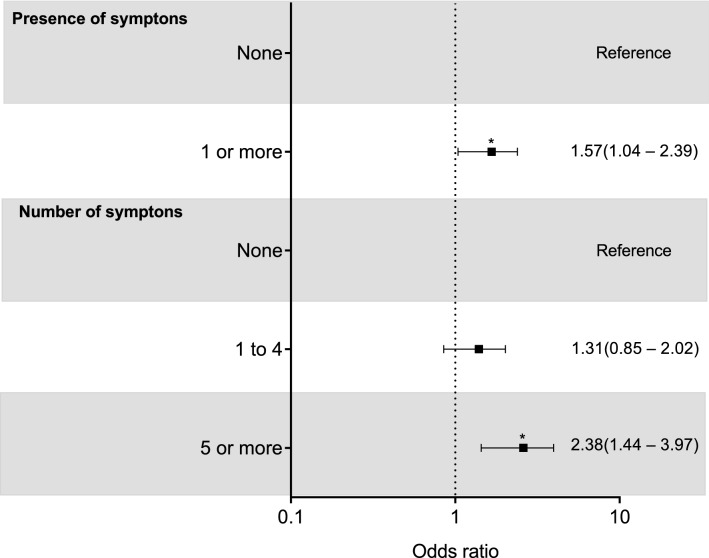
Multivariate-adjusted logistic regression analysis (odds ratio [(95% CI]) of the association between presence and number of persistent symptoms related to COVID-19 (i.e., none, 1–4 and ≥ 5 symptoms) with physical inactivity (< 150 min/week of moderate-to-vigorous activity). *indicates *P* < 0.05;

The adjusted model controlling for confounders (i.e., age [< 60 and ≥ 60 years old], sex [male or female], intensive care unit admission [yes or no], invasive mechanical ventilation [yes or no], hospital length of stay [< 15 and ≥ 15 days], hypertension [yes or no], type 2 diabetes [yes or no], and obesity [BMI < 30 or BMI ≥ 30]) showed that patients with one or more persistent symptoms have greater odds of being physically inactive than those who did not experience any persistent symptoms (OR: 1.57 [95% CI 1.04–2.39], *P* = 0.032) (Fig. 1). In addition, patients reporting 5 or more persistent symptoms showed greater odds of being physically inactive than those without persistent symptoms (OR: 2.38 [95% CI 1.44–3.97], *P* = 0.001) (Fig. 1).

**Figure 2:**
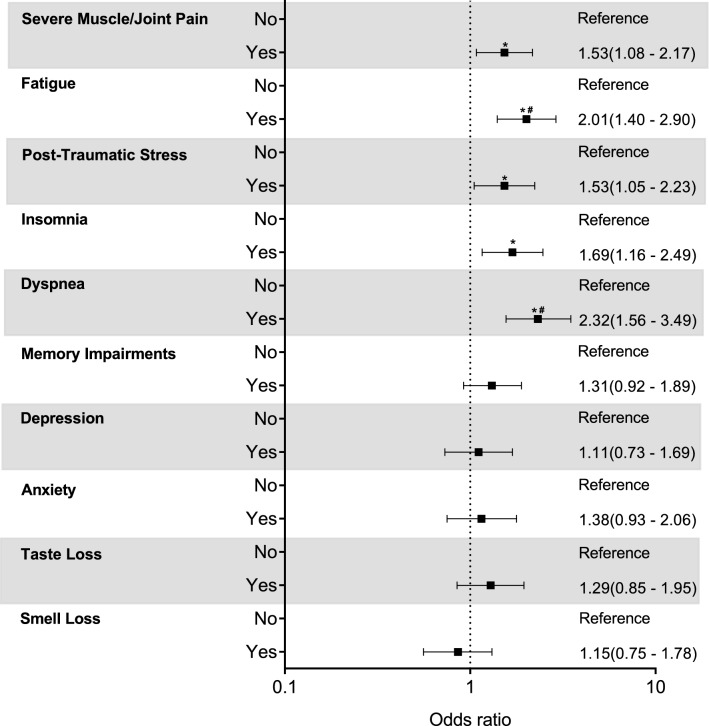
Multivariate-adjusted logistic regression analyses (odds ratio [(95% CI]) of the association between persistent symptoms related to COVID-19 (Severe muscle/joint pain, fatigue, post-traumatic stress, insomnia, dyspnea, memory impairments, depression, anxiety, taste loss, and smell loss) with physical inactivity (< 150 min/week of moderate-to-vigorous activity). *Unadjusted P < 0.05; **#** adjusted *P* < 0.005 (Bonferroni correction).

Adjusted models also showed that severe muscle/joint pain (OR: 1.53 [95% CI 1.08–2.17], *P* = 0.011), fatigue (OR: 2.01 [1.40–2.90], *P* < 0.001), post-traumatic stress (OR: 1.53 [1.05–2.23], *P* = 0.028), insomnia (OR: 1.69 [1.16–2.49], *P* = 0.007), and dyspnea (OR: 2.22 [1.50–3.33], *P* < 0.001) were associated with greater odds of being physically inactive (all *P* < 0.05; Fig. 2). Importantly, fatigue and dyspnea remained as statistically significant predictors of physical inactivity, even after adjusting P-value for multiple comparisons (both *P* < 0.005; Fig. 2). Conversely, memory impairments, depression, anxiety, taste, and smell loss did not significantly associate with physical activity (all *P* > 0.05) (Fig. S1).

## Discussion

The aim of this study was to examine the associations between PASC and physical inactivity in a cohort of COVID-19 survivors (most of them admitted at ICU with pre-existing comorbidities) 6–11 months following hospitalization. The main findings are severalfold: (i) The frequency of physical inactivity was substantive among patients with PASC (60%); (ii) PASC was associated with 57% greater odds of physical inactivity; (iii) the presence of ≥ 5 persistent symptoms vs. none increased the odds of physical inactivity by 138%; (iv). Namely, dyspnea (132%), fatigue (101%), insomnia (69%), post-traumatic stress (53%), and severe muscle/joint pain (53%) were associated with greater odds of physical inactivity. This study provides novel data suggesting that PASC is associated with physical inactivity, which itself may be considered an expected persistent feature among COVID-19 survivors.

There is a growing body of knowledge calling the attention to a high prevalence of PASC worldwide^4–7^. Indeed, a significant proportion of COVID-19 survivors may still present with physical, mental, or cognitive symptoms 6–12 months after the acute infection, particularly in those following ICU treatment^4,14–18^. Whether PASC are risk factors predisposing to a physically inactive lifestyle was so far unexplored.

In our cohort of patients followed 6–11 months after hospitalization in a tertiary hospital, roughly 60% were physically inactive, which exceeds inactivity estimates of 47% for individuals of similar age observed in a population-based study in Brazil^19^. Interestingly, adjusted models suggested that PASC may predispose to physical inactivity, particularly when multiple symptoms are present. We were also able to identify specific symptoms predicting physical inactivity: severe muscle/joint pain, fatigue, post-traumatic stress, insomnia, and dyspnea. Importantly, fatigue and dyspnea remained as significant predictors even after adjusting *P*-value using a highly conservative approach (i.e., Bonferroni correction). These results are of relevance as both fatigue and dyspnea are very frequent PASC and, therefore, may increase the odds to physical inactivity and, ultimately, the risk of poor health outcomes. Some caution should be taken when interpreting these findings, as the design of this study does not allow causative inferences, however plausibility does exist to conjecture that these symptoms, especially when combined, may prevent one from achieving the recommended levels of physical activity.

To the best of our knowledge, this study is the first to investigate associations between individual PASC symptoms with physical inactivity. The adjusted regression models showed that not all PASC symptoms were associated with physical inactivity. The significant associations between specific PASC symptoms (i.e. fatigue, pain, dyspnea, and insomnia) and reduced physical activity could be mediated by different COVID-related pathologies, including persistent pulmonary^20^, renal^21^ or cardiovascular^22^ dysfunction. A proportion of PASC cases may also exhibit a form of myalgic encephalomyelitis/chronic fatigue syndrome ^23^, which is directly associated with signs of persistent systemic inflammation^24^ and can potentially lead to hypoactivity. Regarding mental symptoms, the finding that post-traumatic stress was more related to physical inactivity than depression or anxiety is also potentially interesting, indicating that there may be specific psychiatric manifestations that predispose to physical inactivity in PASC.

Independently of the pathophysiological bases underlying the presence of physical inactivity in association with PASC, an inactive lifestyle is a risk factor that has the potential to increase the demand on health systems worldwide, through increasing both the incidence and aggravation of chronic conditions^11^. Moreover, physical inactivity is an independent risk factor strongly associated with increased mortality; estimates using population attributed fractions suggested that physical inactivity can be responsible for 9% of all-cause mortality worldwide^25^. Importantly, distinct clinical populations have demonstrated a sustained decline in physical activity level after hospital discharge^26,27^. For instance, patients with chronic obstructive pulmonary disease hospitalized to treat acute exacerbation showed a reduction of physical activity levels 1 month after hospital discharge, especially those with more pronounced muscle weakness at the end of the hospitalization period^26^.Recently, a study observed a significant decrease in self-reported walking time 6 months after the onset of symptoms of COVID-19^12^. In this scenario, if COVID-19, and notably PASC, can result in sustained physical inactivity, patients’ survival may be also impacted. Given the multiple types of organ system dysfunctions that may contribute to PASC, further studies are warranted to investigate which of those pathologies may most significantly impact on the emergence of PASC-related physical inactivity—an emerging risk factor that may lead to higher rates of morbidity and mortality. Of relevance, the reversal of inactivity has the potential to attenuate physical, mental and cognitive symptoms that encompass PASC. Therefore, early identification of individuals that could benefit from interventions specifically tailored to promote physical activity may be key to mitigate, at least partially, the burden associated with PASC. Further studies are also warranted to investigate the accurate prevalence and prognostic value of physical inactivity among COVID-19 survivors, and the potential role of vaccination (and perhaps other therapies) on the prevention of inactivity, as seen with other PASC symptoms^25^.

This study is not free of limitations. The observational cross-sectional design hampers establishing cause-and-effect relationships as previously noted, and it may lead to reverse causation bias (i.e., physically inactive individuals may also be prone to PASC, such as fatigue, muscle/joint pain, dyspnea etc.). Physical activity levels were assessed through a questionnaire and reflect the week prior to follow-up assessments. Moreover, the use of questionnaire to assess physical activity is prone to recall bias and overreporting.

In conclusion, among a cohort of COVID-19 survivors showing a high frequency of PASC 6–11 months following hospitalization, the number and type of PASC was predictive of physical inactivity. The novel data provided by this study warrant further investigations to ascertain which COVID-related organ system pathologies may most significantly contribute to the emergence of physical inactivity and help in the early identification of recovering COVID-19 patients who might benefit from interventions to combat inactivity. Considering the potential impact of this risk factor on overall morbidity and mortality and, hence, health systems, healthcare professionals and policy makers should be concerned about COVID-related physical inactivity.

## Methods

### Study design and participants

This study is part of HCFMUSP PASC Initiative, which is a prospective, multidisciplinary cohort study of COVID-19 survivors discharged from the largest tertiary hospital of Latin America (Clinical Hospital, School of Medicine of the University of Sao Paulo).

All patients aged ≥ 18 years who had been admitted (for at least 24 h) as inpatients to our hospital due to laboratory-confirmed COVID-19 between March and August 2020 were consecutively invited for a follow-up in-person visit between October 2020 and April 2021. Exclusion criteria were: previous diagnosis of dementia or end-stage cancer, nosocomial COVID-19 infection, living in long-term care facilities or with insufficient mobility to leave home, and suspected reinfection at the time of follow-up assessment. The details on the study protocol and planned measures have been thoroughly described elsewhere^28^.

This study was approved by the local Ethics Committee (Ethics Committee Approval Number (approval numbers: 4.270.242, 4.502.334, 4.524.031, 4.302.745 and 4.391.560) and registered at the Brazilian Registry of Clinical Trials (https://ensaiosclinicos.gov.br/). All patients provided written informed consent before entering the study. This manuscript was reported according to the Strengthening the Reporting of Observational Studies in Epidemiology (STROBE) Statement. Furthermore, all methods were performed in accordance with the relevant guidelines and regulations.

### Data collection

All patients were evaluated between 6 and 11 months following hospitalization. In brief, patients underwent semi-structured interviewing regarding sociodemographic characteristics, occupational history, lifestyle habits (tobacco and physical activity levels), and self-evaluated health and medical history (with emphasis on previous and present comorbidities, cardiopulmonary symptoms, and medication regimen), and completed a multidisciplinary battery of objective physical assessments and laboratory tests conducted by clinicians and trained non-medical research workers (see reference^28^ for details). Smoking status refers to follow-up assessment (6–11 months after hospital discharge), while pre-existing conditions refers to assessments at the time of hospital admission.

Data from interviews, scales and complementary examinations were captured and stored using real-time web-based case report forms developed on a Research Electronic Data Capture (REDCap) system hosted at the hospital^29^. A team of REDCap experts managed the database and provided access for the different research groups to conduct interim and final statistical analyses.

### Physical inactivity

Physical activity was assessed during the in-person follow-up visits by experienced researchers using The International Physical Activity Questionnaire-Short Form (IPAQ). In brief, IPAQ inquiries about physical activity in the past 7 days. Time spent in each activity was calculated as the number of days multiplied by the number of hours reported. Participants were classified as physically inactive according to WHO Guidelines (i.e., < 150 min/week of moderate-to-vigorous intensity physical activity).

### Post-acute sequelae of SARS-CoV-2 infection

For the present investigation, we used data regarding ten self-reported symptoms deemed as relevant to PASC^9,30^ which were evaluated using standardized scales applied by specialized teams during the in-person visits, including: post-traumatic stress disorder^31^, anxiety and depression^32^, insomnia^33^, subjective memory impairment^34^, fatigue^35^, dyspnea^36^, severe muscle/joint pain^36^, and taste and smell loss)^37^. For all dependent variables, validated scale cutoffs were used to generate categorical ‘yes–no’ variables. For all variables but post-traumatic stress, subjects were asked about the presence of symptoms before hospitalization, in order to confirm that the onset of symptoms occurred after COVID-19.

### Statistical analyses

Characteristics of patients 6–11 months following hospitalization are presented as absolute (n) and relative (%) frequency. The association of the outcome of interest (physical inactivity) was assessed by means of multivariable logistic regression adjusted by age [< 60 and ≥ 60 years old], sex [male or female], intensive care unit admission [yes or no], invasive mechanical ventilation [yes or no], hospital length of stay [< 15 and ≥ 15 days] and pre-existing conditions (hypertension [yes or no], type 2 diabetes [yes or no], and obesity [BMI < 30 or BMI ≥ 30]). Confounders were selected based on a Direct Acyclic Graph (DAG, www.dagitty.net), which is a causal diagram based on causal relations between the exposure, outcome, and potential confounders^38^. The DAG was developed from a priori knowledge to identify a minimum yet sufficient set of covariates to remove confounding factors from the statistical analysis^39^ (Fig. S1). Odds ratios were calculated along their corresponding 95% confidence intervals (95% CI). For associations between each PASC (i.e., post-traumatic stress disorder, anxiety and depression, insomnia, memory impairment, fatigue, dyspnea, severe muscle/joint pain, and taste and smell loss) and physical inactivity, significance level was set at *P* ≤ 0.005 (according to Bonferroni correction for multiple tests). All other significance levels were set at *P* ≤ 0.05. All analyses were performed in the statistical environment R (version 3.5.3; R Core Team 2020).

## Supplementary Information

Supplementary Information.

## Data Availability

All background information on individuals and clinical information for patients included in this study are available from corresponding author on reasonable request.
