# Supplementary material for: Post-acute sequelae of SARS-CoV-2 associates with physical inactivity in a cohort of COVID-19 survivors
Source: Sci Rep. 2023 Jan 5;13:215. doi: 10.1038/s41598-022-26888-3 (PMC9813883; doi:10.1038/s41598-022-26888-3)

SUPPLEMENTARY MATERIAL

**Figures and legends**

Figure S1. Direct acyclic graph of the association between post-acute sequelae of SARS-CoV-2 (PASC) and physical activity level. BMI = body mass index; ICU = Intensive Care Unit admission; IMV = Invasive Mechanical Ventilation.

**Figure S1**


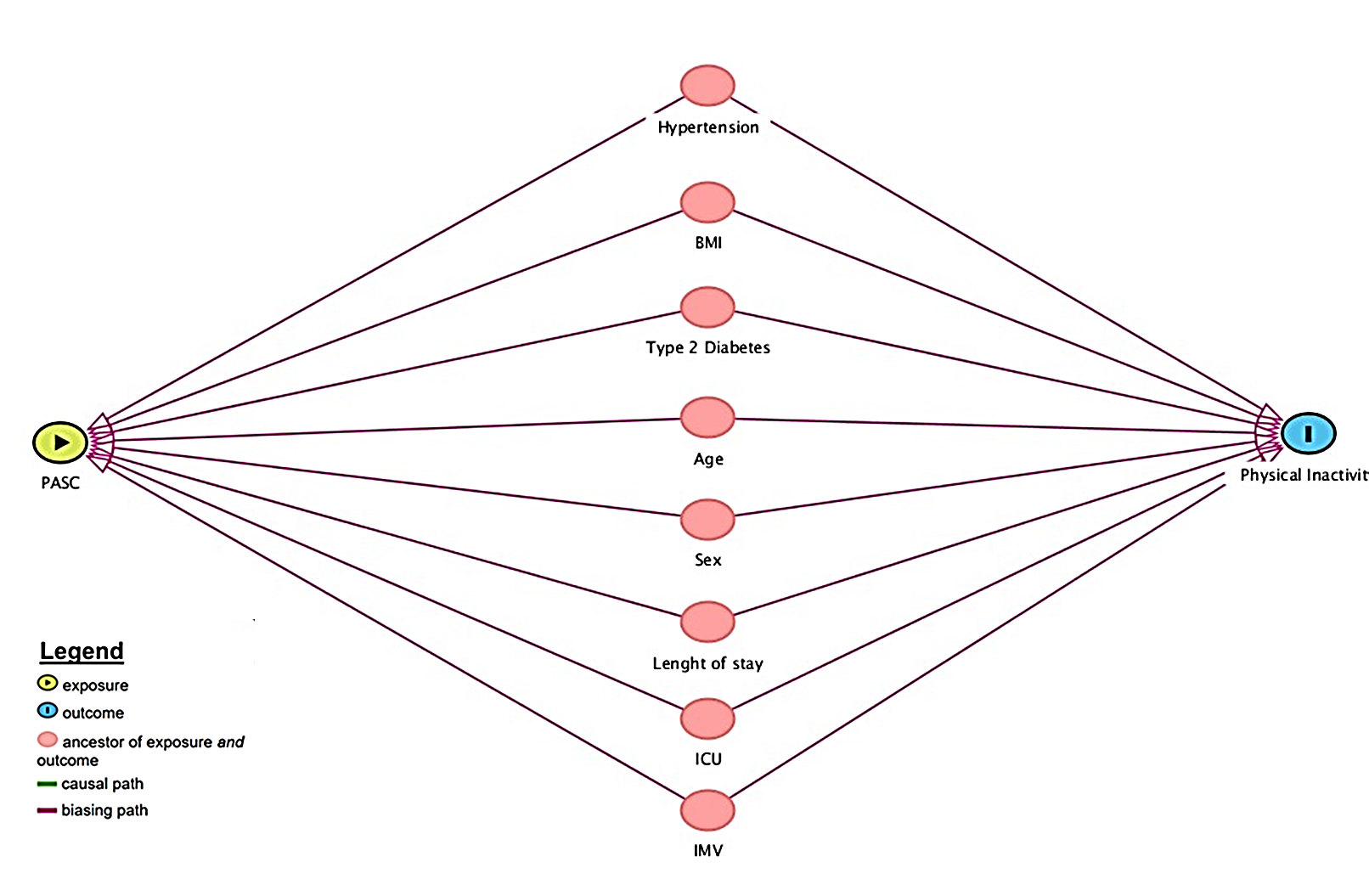

Supplement: Supplementary file 1 — Supplementary Information. [file 41598_2022_26888_MOESM1_ESM.docx]
